# Supplementary material for: Comprehensive proteogenomic characterization of early duodenal cancer reveals the carcinogenesis tracks of different subtypes
Source: Nat Commun. 2023 Mar 29;14:1751. doi: 10.1038/s41467-023-37221-5 (PMC10060430; doi:10.1038/s41467-023-37221-5)
Supplement: Supplementary file 1 — Supplementary Information [file 41467_2023_37221_MOESM1_ESM.pdf]

Supplementary Fig. 1

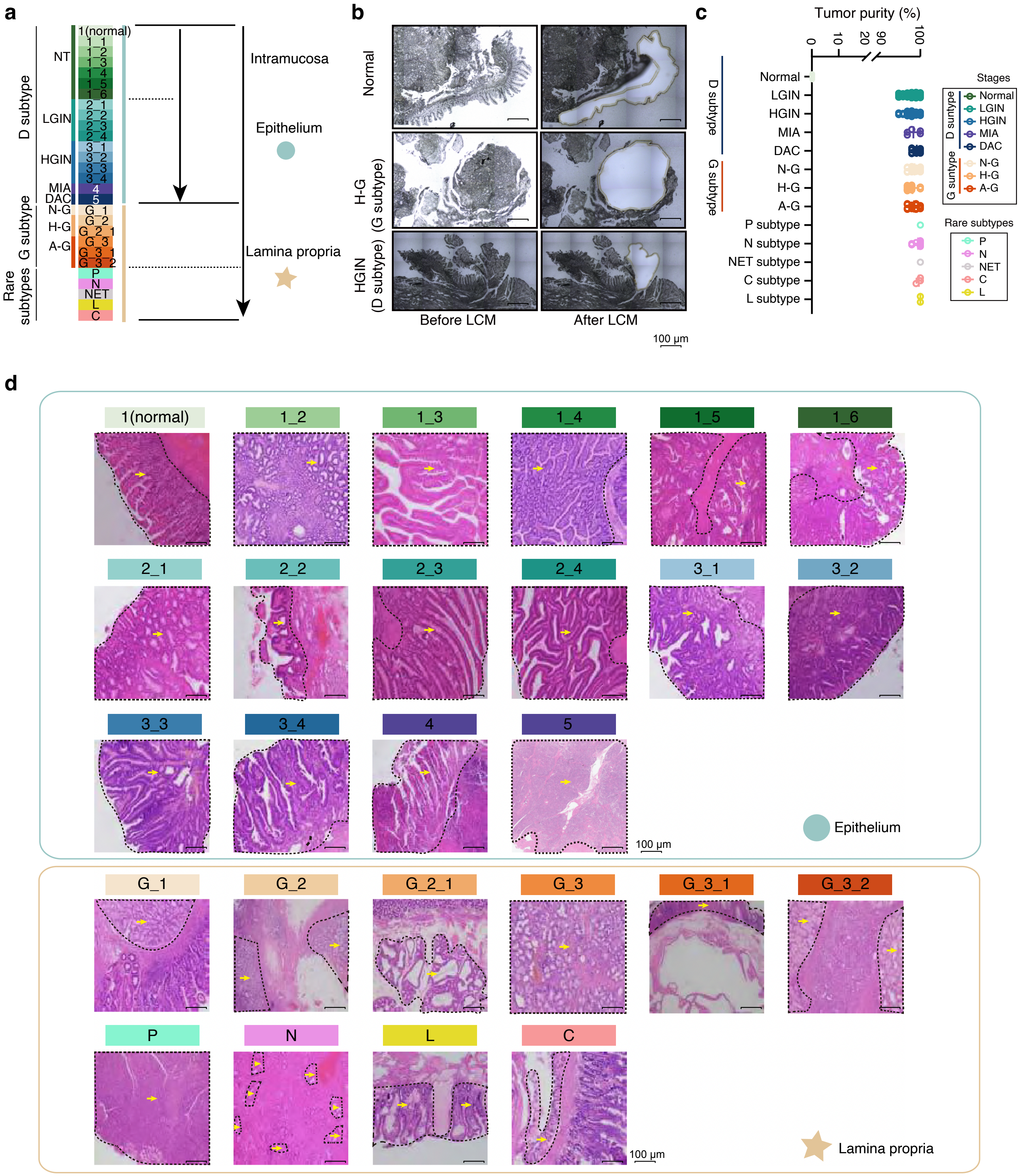

Supplementary Fig. 2

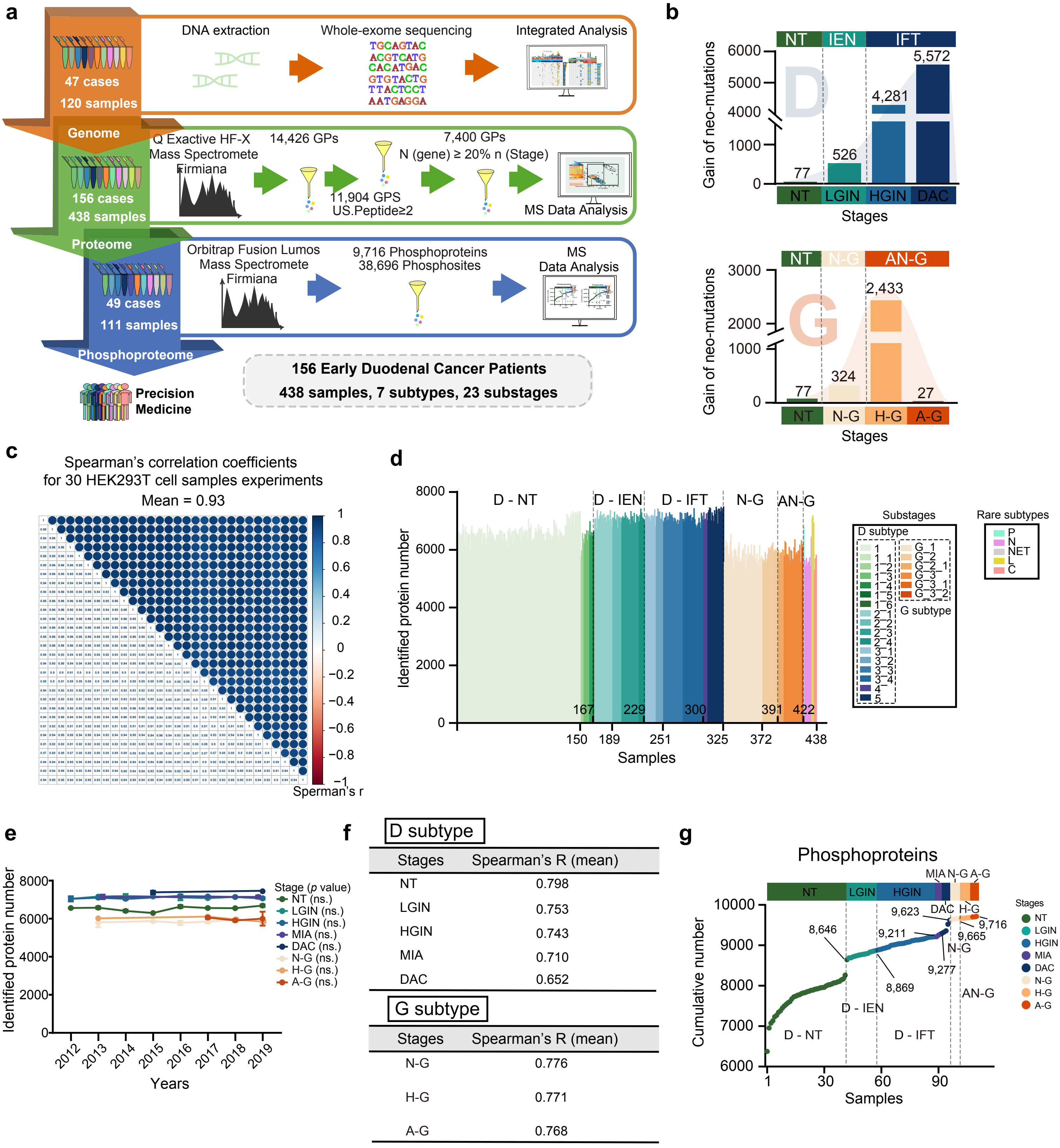

D subtype

Stages	Spearman's R (mean)
NT	0.798
LGIN	0.753
HGIN	0.743
MIA	0.710
DAC	0.652

G subtype

Stages	Spearman's R (mean)
N-G	0.776
H-G	0.771
A-G	0.768

Supplementary Fig. 3

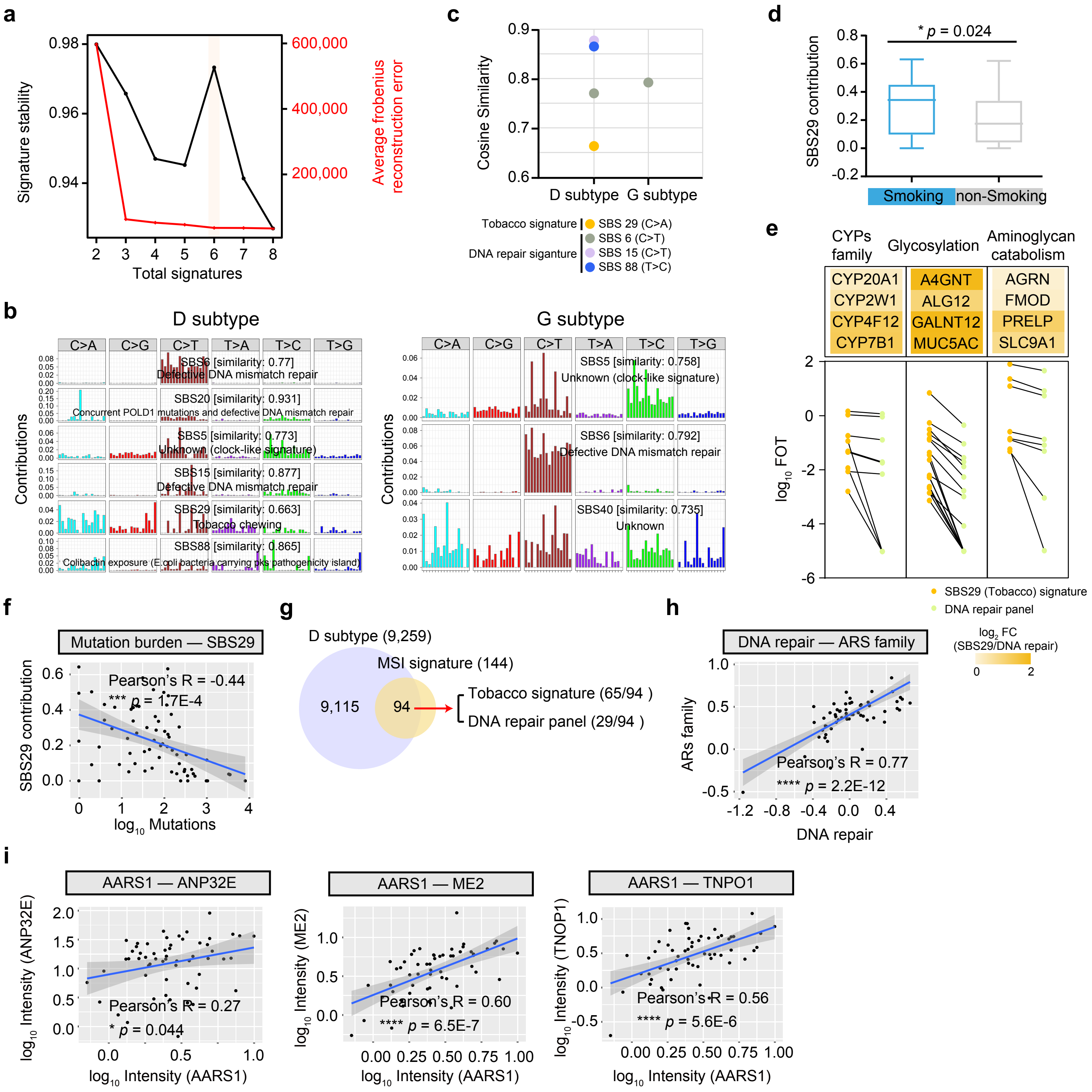

Supplementary Fig. 4

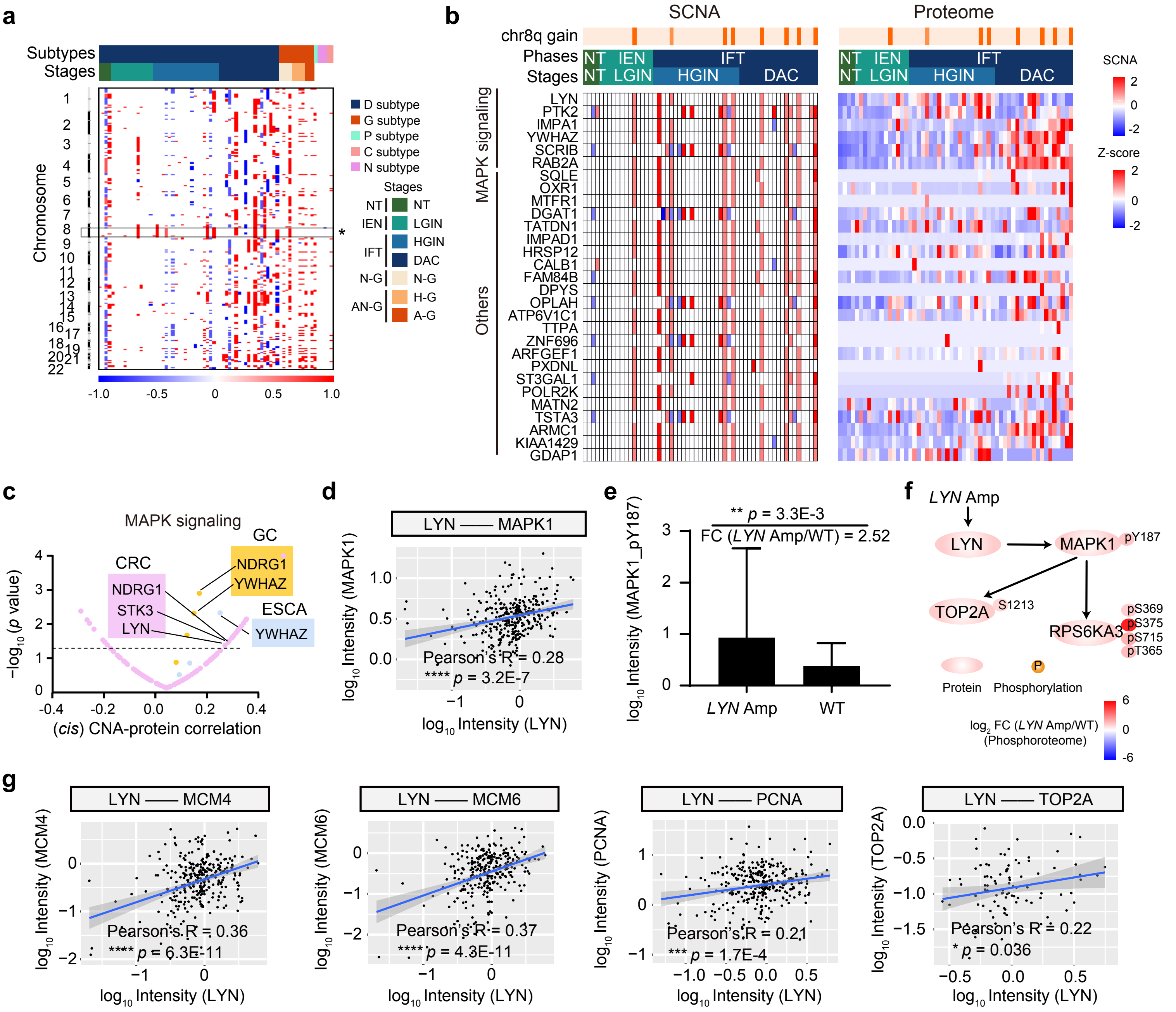

Supplementary Fig. 5

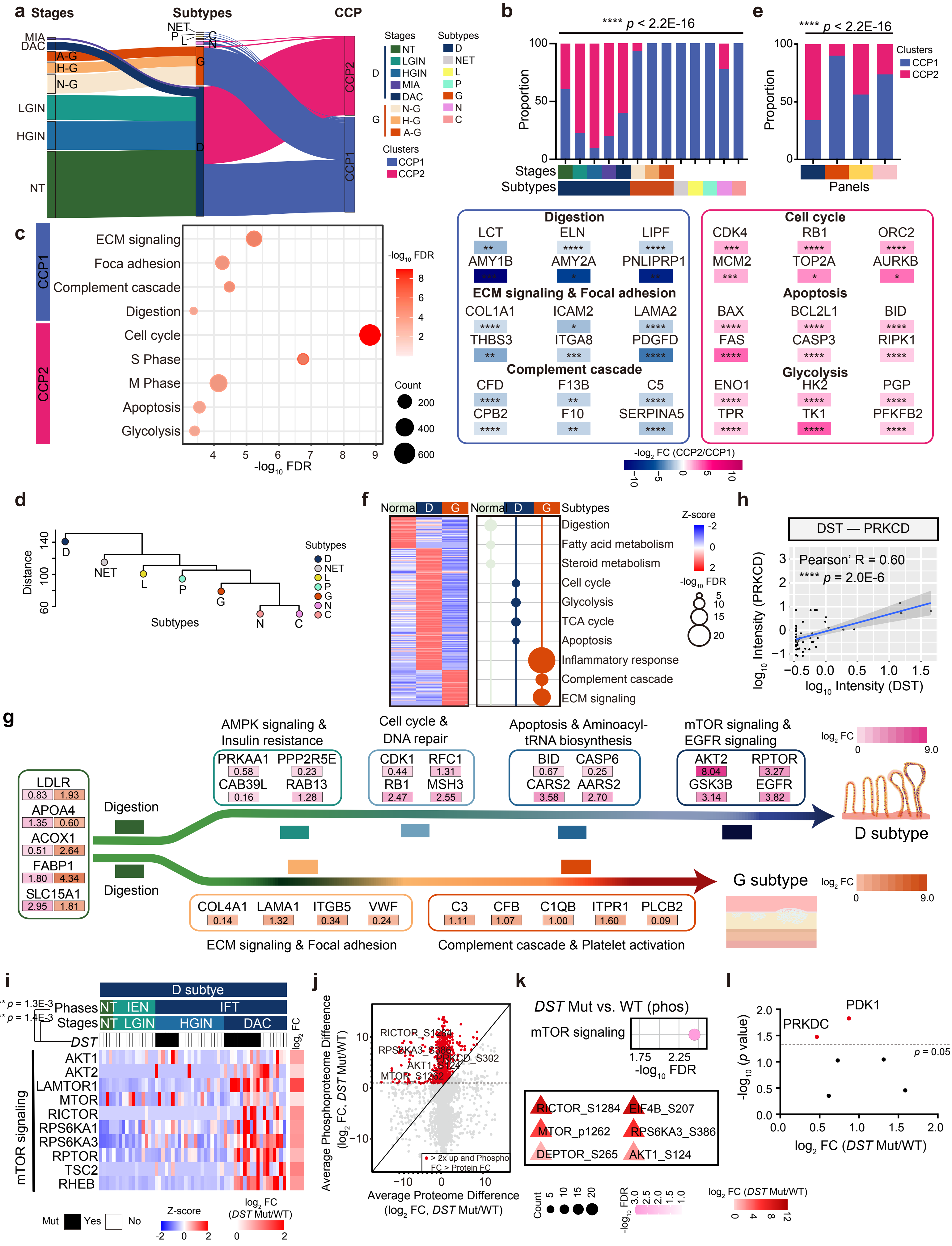

Supplementary Fig. 6

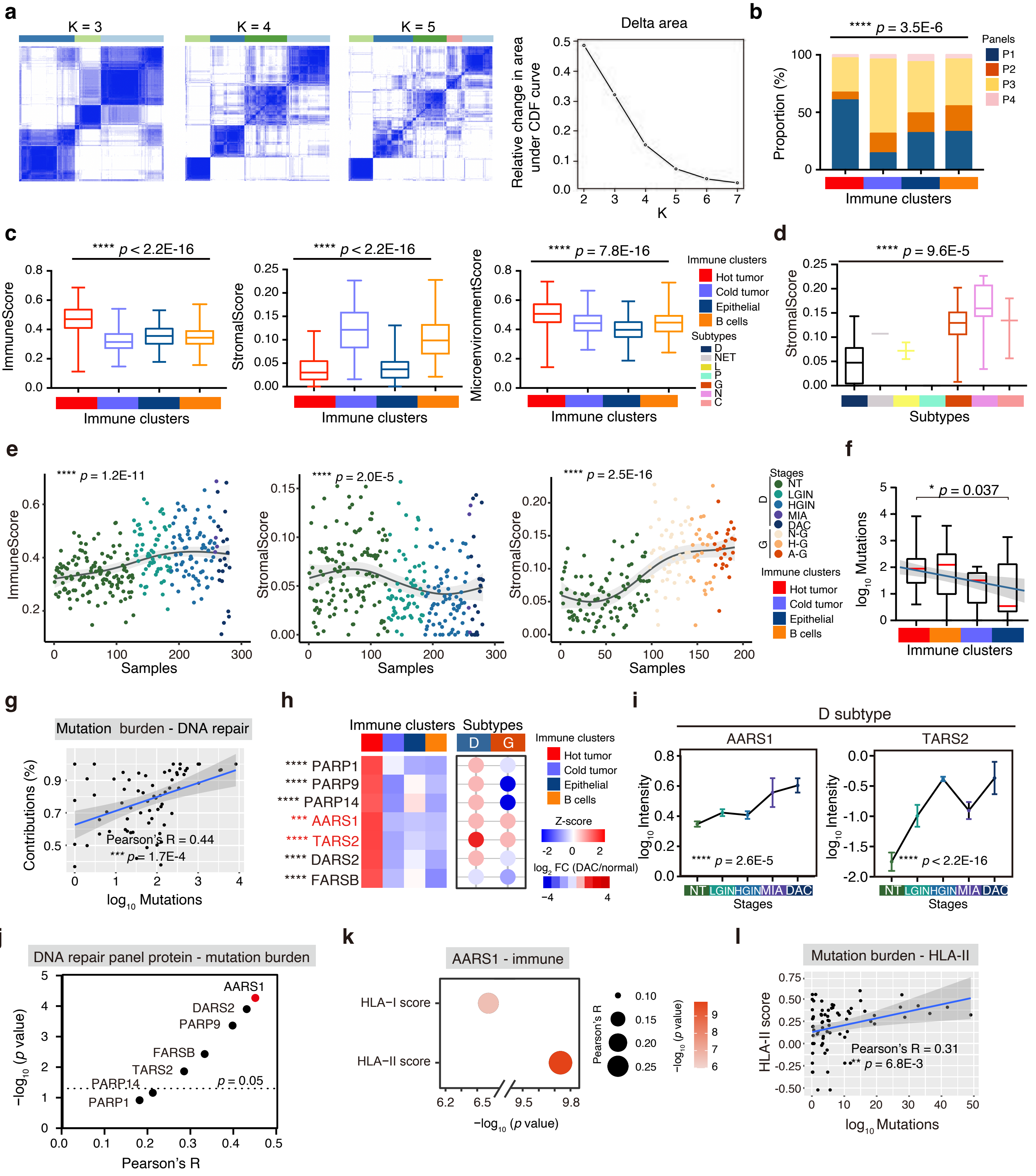

Supplementary Fig. 7

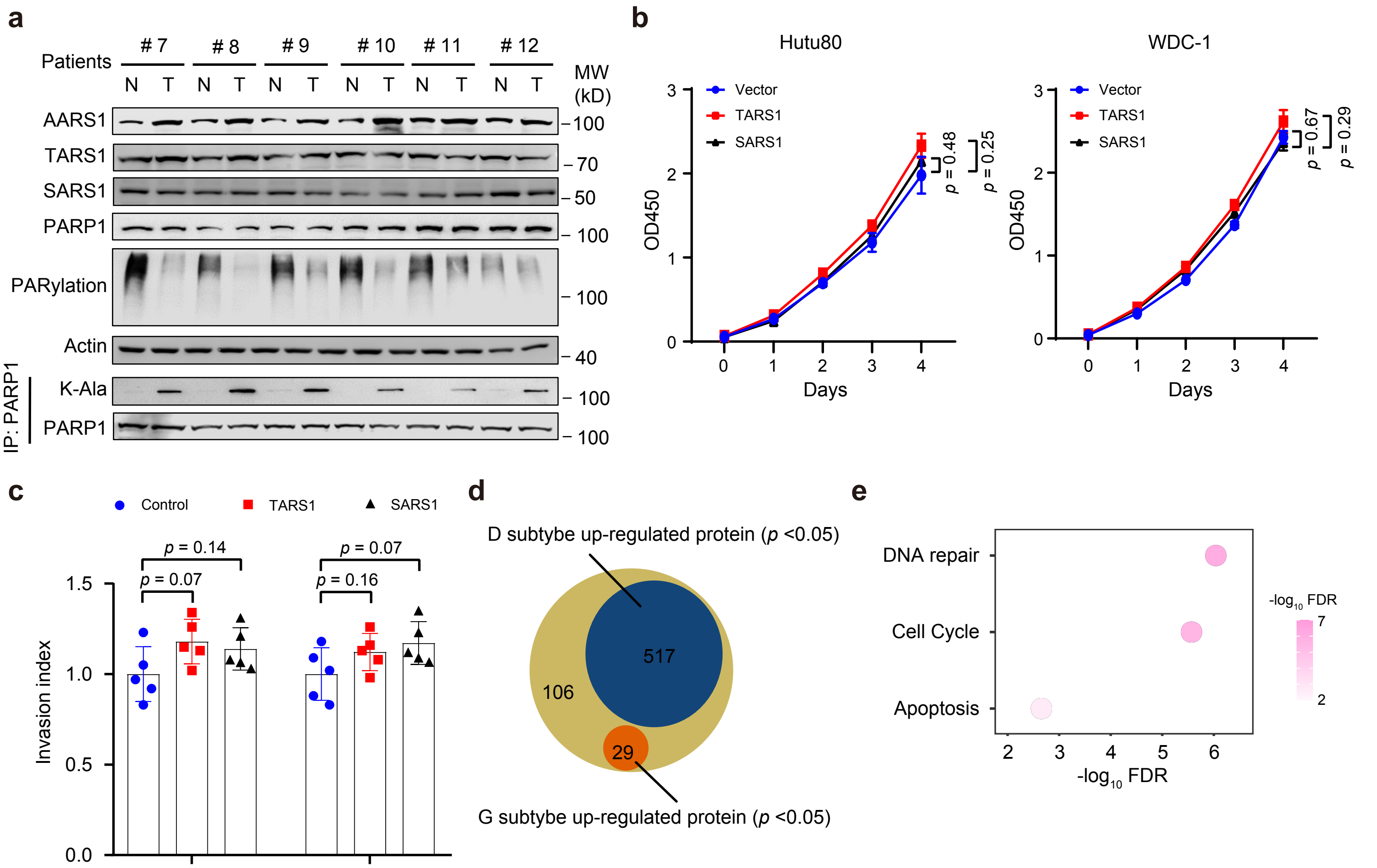

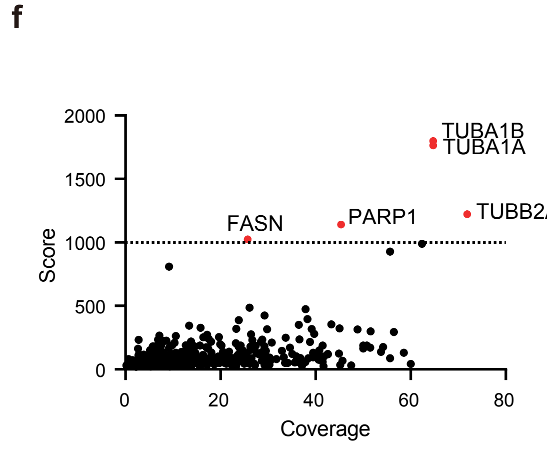

g

TAP		
Protein	Score	Coverage
PARP1	1968.2	60.24
TOP1	1858.55	60.00
LIG3	1470.75	58.18
PARP2	1175.64	65.91
PRKDC	867.23	51.96
XRCC6	847.57	51.94
XRCC5	635.97	48.11
XRCC1	430.55	34.91

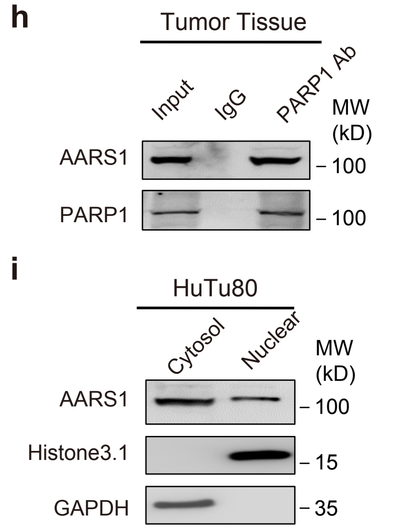

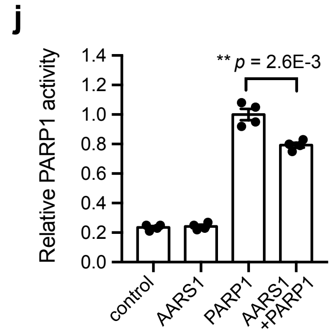

Supplementary Fig. 8

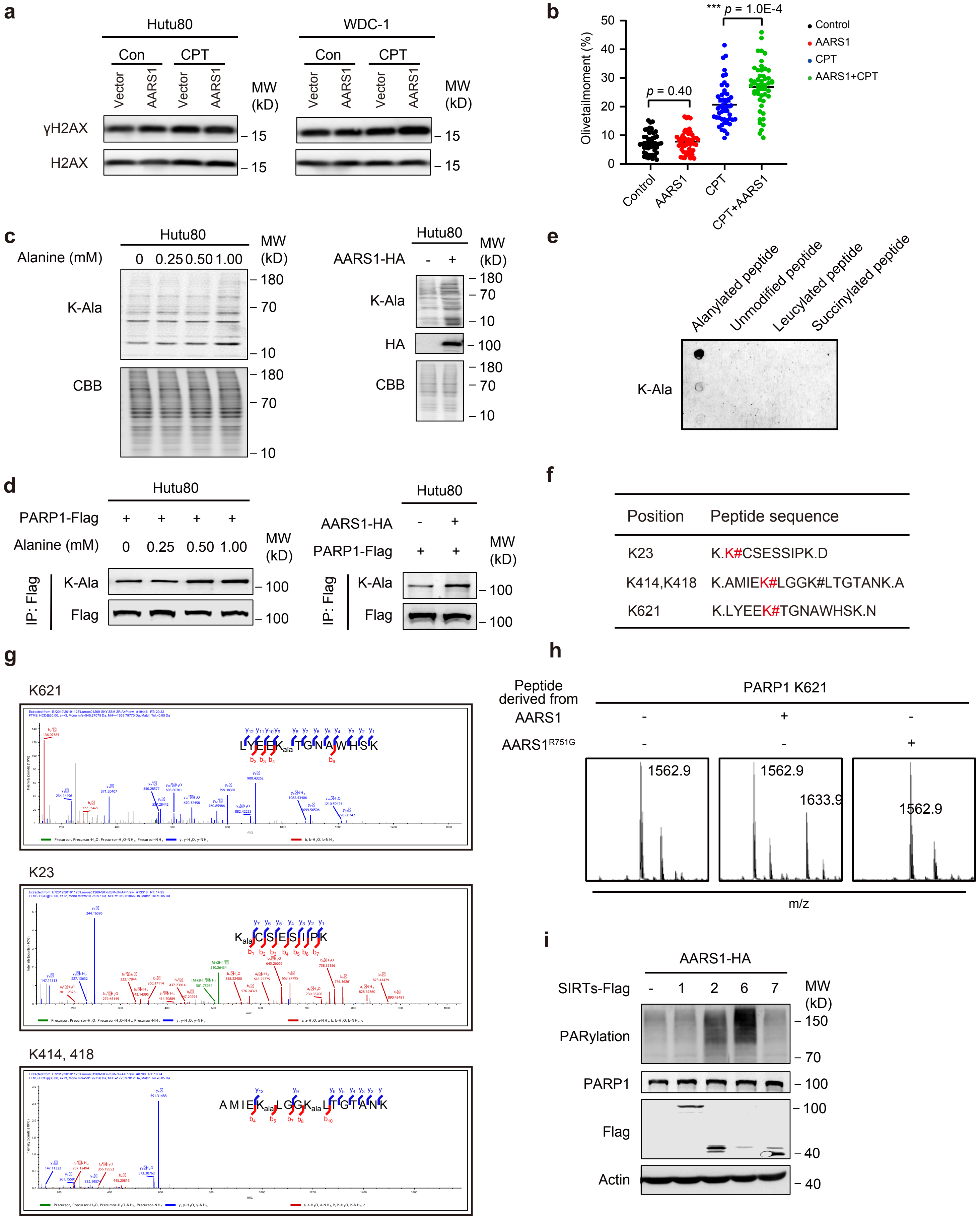

Supplementary Fig. 9

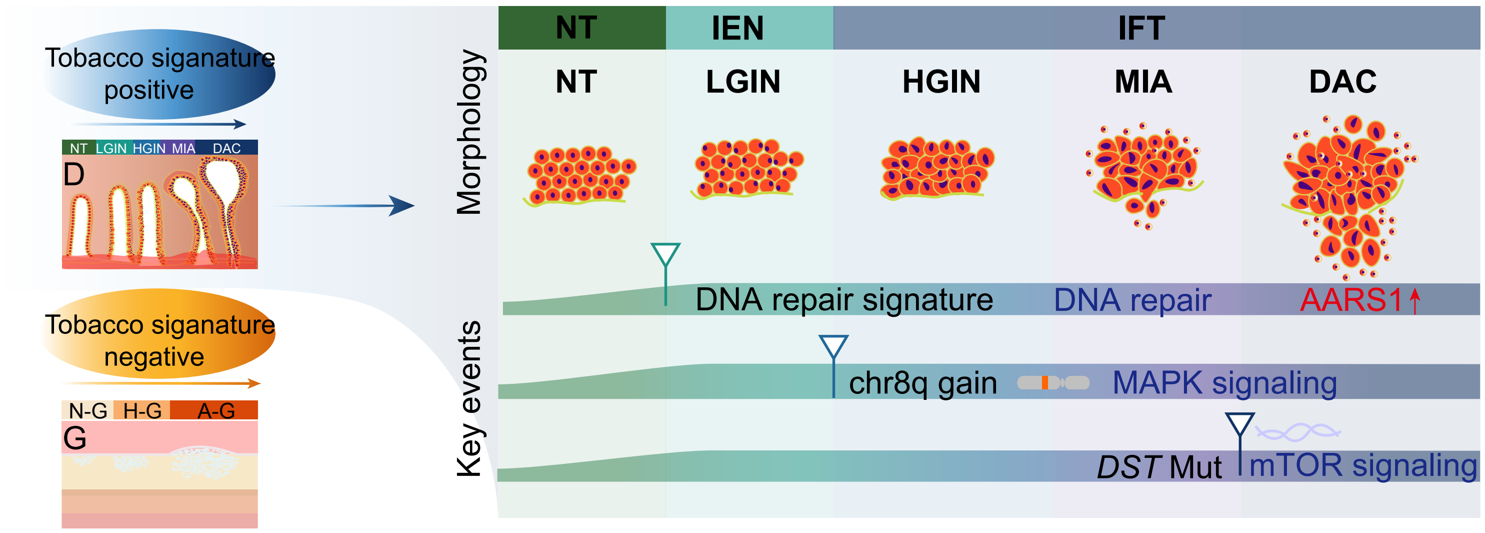

Supplementary Figure Legends

Supplementary Fig. 1 | The pathological characteristics of the samples in DC. a, The pathological characteristics and invasion layer of the D and G subtypes. **b**, The microdissection of stage compartments of DC samples. The scale bar indicates 100 μm. **c**, Boxplot showing tumor purity of the samples in DC (mean ± SD). n (normal) = 150, n (LGIN) = 62, n (HGIN) = 71, n (MIA) = 5, n (DAC) = 20, n (N-G) = 47, n (H-G) = 26, n (A-G) = 24, n (P subtype) = 1, n (N subtype) = 9, n (NET subtype) = 1, n (C subtype) = 3, n (L subtype) = 2 biologically independent samples examined. Boxplot shows median (central line), upper and lower quartiles (box limits), 1.5× interquartile range (whiskers). **d**, HE staining of the components in DC. The yellow arrows indicate the related tissue, and the dot/arrows show the pathological characterization of the substages and subtypes. The thickness of the FFPE slide is 3 μm. HE: hematoxylin and eosin. The scale bar indicates 100 μm.

Supplementary Fig. 2 | Overview of the proteomic profiles of early and progressive DC cohorts. a, The workflow of sampling, processing, and analysis of early-stage DC at the multi-omics level. The 438 samples included 2 major and 5 rare subtypes. **b**, The number of the gain of neo-mutations in the D (left) and G (right) subtypes progression. **c**, (Spearman's) Correlation analysis of 30 HEK293T cell samples as MS quality control to evaluate the robustness of label-free quantification. **d**, The identification protein numbers of 438 samples. **e**, Boxplot showing the number of protein identifications of the samples spanning from 2012 to 2019 at the same pathological stage (Kruskal-Wallis test). Boxplot shows median (central line), upper and lower quartiles (box limits), 1.5× interquartile range (whiskers). **f**, Table showing Spearman's correlation coefficients of stages in the carcinogenesis of the D (left) and G (right) subtypes. **g**, The cumulative number of phosphoprotein identifications in DC progression. **** $p < 1.0E-4$, *** $p < 1.0E-3$, ** $p < 0.01$, * $p < 0.05$, ns. > 0.05. Source data are provided as a Source data file.

Supplementary Fig. 3 | The specific characterization and mutational signature of DC. a, Identifying the number of processes operating in a set of DC samples (n = 120) based on the reproducibility of their signatures and average Frobenius reconstruction error. **b**, The dominant signatures and the corresponding cosine similarity in the D (left) and G (right) subtypes. **c**, The major somatic SNVs signature in the D and G subtypes. **d**, Boxplot presenting the SBS29 contribution in the patients with the habit of smoking or not (two-sided Wilcoxon signed-rank test). n (smoking) = 15, n (not smoking) = 53 biologically

independent samples examined. Boxplot shows median (central line), upper and lower quartiles (box limits), 1.5× interquartile range (whiskers). **e**, The represented proteins from CYPs and the predominant pathways of the Tobacco signature. n (Tobacco signature) = 21, n (DNA repair signature) = 47 biologically independent samples examined. **f**, Scatterplot showing the relationship between the Tobacco signature and mutation burden (two-sided Pearson's correlation test). **g**, The number of the MSI related proteins in the Tobacco signature and the DNA repair panel. **h**, Scatterplots showing the relationship between DNA repair and ARS family at the protein level (two-sided Pearson's correlation test). ARS: Aminoacyl-tRNA synthetases**. i**, Scatterplot showing the relationship between AARS1 and MSI related proteins at the protein level (two-sided Pearson's correlation test). Error bars represented mean ± SEM. **** p < 1.0E-4, *** p < 1.0E-3, ** p < 1.0E-2, * p < 0.05, ns. > 0.05. Source data are provided as a Source data file.

Supplementary Fig. 4 | The amplification of *LYN* has positive impacts on MAPK signaling and cell proliferation. a, Profiling of absolute CNAs observed in DC. The square directs to a subset of patient samples used for WES (n = 120). **b**, The *cis* SCNA-protein regulations of correlated genes (left) on their corresponding proteins expression (right). The square directs to a subset of patient samples in the D subtype used for WES (n = 120). **c**, Volcano plot showing the *cis*- correlation of the SCNA (x axis) at chr8q gain and the associated -$\log_{10}$ (p value) (y axes) on the corresponding proteins of TCGA cohort (two-sided Spearman's correlation test). **d**, Scatterplot showing the relationship between $\log_{10}$ LYN and $\log_{10}$ MAPK1 expression at the protein level (two-sided Pearson's correlation test). **e**, Column showing the expression of MAPK Y187 in the *LYN* amplification group and WT group (two-sided Wilcoxon signed-ranked test, mean ± SD). **f**, A brief summary showing the regulation of LYN on the downstream substrates. **g**, Scatterplots showing the relationship between $\log_{10}$ LYN and cell proliferation related markers at the protein level (two-sided Pearson's correlation test). **** p < 1.0E-4, *** p < 1.0E-3, ** p < 0.01, * p < 0.05, ns. > 0.05. Source data are provided as a Source data file.

Supplementary Fig. 5 | The association between proteomic clusters and subtype-based panels, and dynamic driver pathway waves of proteomic panels in DC progression. a, Sankey diagram analysis of all 438 samples from 156 cases classified into two proteomic clusters. **b,** Histogram showing the proportions of the proteomic clusters in DC subtypes (two-sided Fisher's exact test). **c**, The represented pathways (left) and related proteins (right) in the CCP1 and CCP2 (two-sided Wilcoxon signed-rank test).

d, Hierarchical clustering analysis of 2 major and 5 rare subtypes of DC. **e,** Histogram showing the proportions of the proteomic clusters in the panels (two-sided Fisher's exact test). **f**, The represented pathways of the normal tissues, D subtype, and G subtype in the P3. **g**, The staging molecular models drove carcinogenesis from early to progressive D (top) and G (bottom) subtypes. **h**, Scatterplot showing the relationship between $\log_{10}$ DST and $\log_{10}$ PRKCD at the protein level (two-sided Pearson's correlation test). **i**, Heatmap showing the *DST* mutation positive associated proteins in mTOR signaling (two-sided Wilcoxon signed-rank test, BH-adjusted $p < 0.05$). **j**, Fold changes of proteins and phosphosites, and their correlations in the *DST* mutation group and WT group (two-sided Wilcoxon signed-ranked test). Red dots: phosphosites are greater than twofold changes in the *DST* mutation group vs. WT group, and changes of phosphosites abundance are greater than changes of their corresponding protein abundance. **k**, The represented pathways in the *DST* Mut group at the phosphoprotein level. **l**, Volcano plot showing the impacts of *DST* mutation on the protein-levels of kinases (two-sided Wilcoxon signed-ranked test). **** $p < 1.0E-4$, *** $p < 1.0E-3$, ** $p < 1.0E-2$, * $p < 0.05$, ns. > 0.05. Source data are provided as a Source data file.

Supplementary Fig. 6 | Immune-based clustering of DC. a, Immune-based consensus clustering of DC samples. **b**, The proportion of panels in the four immune clusters (two-sided Fisher's exact test). **c**, Boxplots showing the immune infiltration in the four immune clusters (Kruskal-Wallis test). Boxplots show median (central line), upper and lower quartiles (box limits), 1.5× interquartile range (whiskers). **d**, Boxplot showing stroma score in the 2 major and 5 subtypes in DC (Kruskal-Wallis test). Boxplot shows median (central line), upper and lower quartiles (box limits), 1.5× interquartile range (whiskers). **e**, Scatterplots presenting the immune infiltration in the D and G subtypes progression (Kruskal-Wallis test). **f**, Boxplot showing the mutation burden in the four immune clusters (Kruskal-Wallis test). Shading showing 95% confidence intervals for the slopes. Boxplot shows median (central line), upper and lower quartiles (box limits), 1.5× interquartile range (whiskers). **g**, Scatterplot showing the positive correlation between DNA repair panel and the mutation burden (two-sided Pearson's correlation test). **h**, The expression of the significant molecules of the DNA repair panel in the four immune clusters (Kruskal-Wallis test) (left) and in the tumor tissues of the D and G subtypes (right). **i**, The expression of AARS1/TARS2 in the D subtypes (Kruskal-Wallis test, mean ± SEM). **j**, The (Pearson's) correlation between the significant molecules of the DNA repair panel and the mutation burden (two-sided Pearson's

correlation test). **k**, Scatterplot displaying the (Pearson's) correlation between AARS1 and HLA-I/II score at the protein level. **l**, Scatterplot showing the relationship between the mutation burden and HLA-II (two-sided Pearson's correlation test). **** $p < 1.0E-4$, *** $p < 1.0E-3$, ** $p < 0.01$, * $p < 0.05$, ns. > 0.05. Source data are provided as a Source data file.

Supplementary Fig. 7 | Increased AARS1 contributes to the development of DC. **a**, Western blot analysis of AARS1, TARS1, SARS1, PARP1, PARylation, actin, and lysine alanylation of PARP1 in tumor tissues (T) and tumor-adjacent normal tissues (N). **b**, The impacts of TARS1/SARS1 overexpressed (OE) on Hutu80 cells (left) and WDC-1 cells' (right) proliferation (n = 5 repeats per group) (two-sided Student's t-test, mean ± SD): vector (control), OE-TARS1, and OE-SARS1. The OD450 value and days were shown on the y and x axes, respectively. **c**, The impacts of TARS1/SARS1-OE on Hutu80 cells (left) and WDC-1 cells (right) invasion (n = 5 repeats per group) (two-sided Student's t-test, mean ± SD). **d**, Venn diagram showing the overrepresented proteins from 652 proteins in the D and G subtypes (Kruskal-Wallis test, BH-adjusted $p < 0.05$). **e**, Gene Ontology enrichment analysis displaying the represented pathways of the overrepresented proteins from 652 proteins in the D subtype. **f**, Scatterplot presenting the proteins involved in DNA damage repair exhibit strong interactions with AARS1. **g**, Proteins involved in DNA damage repair exhibit strong interactions with AARS1. **h**, Co-immunoprecipitation showing that endogenous AARS1 interacted with endogenous PARP1. **i**, Cellular localization of AARS1. **j**, The relative reduction of PARP1 activity by interacting with AARS1 (n = 4 repeats per group, two-sided Student's t-test, mean ± SD). **** $p < 1.0E-4$, *** $p < 1.0E-3$, ** $p < 0.01$, * $p < 0.05$, ns. > 0.05. Source data are provided as a Source data file.

Supplementary Fig. 8 | AARS1 inhibits PARP1 activity with PARylation. **a**, Western blot analysis of γH2AX and H2AX. **b**, Comet assay detection of DNA damage levels (two-sided Student's t-test, mean ± SD). For each group, the DNA damage levels from a total of 30 cells from five independent repeats were measured. **c**, The impacts of alamine (left) and AARS1 (right) on the K-Ala levels of total protein in Hu80 cells. **d**, The effects of PARP1-alamine and AARS1-PARP1 on the K-Ala levels in Hutu80 cells. **e**, Dot blot analysis showing that the K-Ala antibody reacts specifically with an alanylated peptide. **f**, The K-Ala sites in the PARP1 protein. Amino acid sequences are shown, and modified lysine residues were indicated by #. **g**, The full MS/MS spectra are shown the identification of K621, K23, and K414, 418 Ala modification sites of PARP1 in cells. **h**, Purified AARS1 and enzymatic defective $AARS1^{R751G}$

are tested for their capacities to catalyze K-Ala formation in the synthetic K621 peptide. **i**, The effects of SIRTs on the cellular PARylation in Hutu80 cells. **** $p < 1.0E-4$, *** $p < 1.0E-3$, ** $p < 0.01$, * $p < 0.05$. Source data are provided as a Source data file.

Supplementary Fig. 9 | The model of the key events in the progression of DC.
