# Supplementary material for: Comprehensive proteogenomic characterization of early duodenal cancer reveals the carcinogenesis tracks of different subtypes
Source: Nat Commun. 2023 Mar 29;14:1751. doi: 10.1038/s41467-023-37221-5 (PMC10060430; doi:10.1038/s41467-023-37221-5)
Supplement: Supplementary file 3 — Description to Additional Supplementary Information [file 41467_2023_37221_MOESM3_ESM.pdf]

Description of Supplementary Dataset:

File Name: **Supplementary Data 1**

Description: **Clinical Characteristics of DC Patients. a**, The information of 156 cases (438 samples) for proteomic profiling; each sample has a specific experiment ID in firmiana paltform. **b**, The tumor purity of 438 samples in DC. **c**, Clinical characteristics of 49 cases (111 samples) for phosphoproteomics profiling; each sample has a specific experiment ID in firmiana paltform.

File Name: **Supplementary Data 2**

Description: **Somatic Mutations and Copy Number Alterations of 120 Samples for WES in DC Progression. a**, The coverage depth and rawdata of 120 samples of DC. **b**, The mutational information of 120 samples of DC. **c**, Somatic copy number alterations of 120 samples in DC progression.

File Name: **Supplementary Data 3**

Description: **The Identifications of Proteins and Phosphosites in DC, and Spearman's Correlation of 30 HEK293T Cell Samples. a**, The correlations analysis of 30 HEK293T cell samples as quality control. **b**, The proteins identification (11,904) by screened with high confidence unique proteins identified with ≥2 unique strict peptides and high abundance range (FOT ≥1.0E-5). Note: The NA and 0 were changed by 1.0E-5. **c**, The (normalized) phosphosites identification of 111 samples in DC (identified in more than 20% samples of every substage).

File Name: **Supplementary Data 4**

Description: **The Score of the Two Major Somatic Signatures of DC and the Cancer Associated Genes. a**, The score of the two major somatic sigantures of DC. **b**, The list of the cancer associated genes.

File Name: **Supplementary Data 5**

Description: **The Immune Infiltration of DC Subtypes. a**, The immune-/stroma-/microenvironment- Score and the cell type compositions of 438 samples across four immune clusters.

File Name: **Supplementary Data 6**

Description: **AARS1 Promoted Cancer Cell Proliferation through Non-canonical Function, and Lysine Alanylation of PARP1 Decreased Cell Apoptosis by Inhibiting PARP1 in DC. a**, More expression of AARS1 and PARP1 in tumor tissue was detected by western blot analysis. **b**, The impacts of AARS1 overexpressed (OE) on Hutu80 cells and WDC-1 cells proliferation (n = 5 repeats per group). **c**, The impacts of AARS1 knock-down on Hutu80 cells and WDC-1 cells proliferation (n = 5 repeats per group). d, The effects of AARS1 OE (left)/knock-down (right) on Hutu80 cells and WDC-1cells invasion (n = 5 repeats per group). e, The impacts of TARS1/SARS1 overexpressed (OE) on Hutu80 cells (left) and WDC-1 cells (right) proliferation (n = 5 repeats per group). f, The impacts of TARS1/SARS1-OE on Hutu80 cells (left) and WDC-1 cells (right) invasion (n = 5 repeats per group). g, Activities of PARP1 immunoprecipitated from cells transfected with different aminoacyl-tRNA synthetases. h, The relative reduction of PARP1 activity by interacting with AARS1. i, The impacts of AARS OE on Hutu80 cells and WDC-1 cells apoptosis (n = 5 repeats per group). j, Western blot analysis of γH2AX, H2AX (top), and

comet assay detection of DNA damage levels. k, DNA binding affinities of PARP1 and PARP1 mutants.
