# Supplementary material for: Comprehensive proteogenomic characterization of early duodenal cancer reveals the carcinogenesis tracks of different subtypes
Source: Nat Commun. 2023 Mar 29;14:1751. doi: 10.1038/s41467-023-37221-5 (PMC10060430; doi:10.1038/s41467-023-37221-5)
Supplement: Supplementary file 10 — Reporting Summary [file 41467_2023_37221_MOESM10_ESM.pdf]

nature portfolio

Corresponding author(s): Chen Ding
Yingyong Hou
Jian-Yuan Zhao

Last updated by author(s): Mar 7, 2023

Reporting Summary

Nature Portfolio wishes to improve the reproducibility of the work that we publish. This form provides structure for consistency and transparency in reporting. For further information on Nature Portfolio policies, see our Editorial Policies and the Editorial Policy Checklist.

Statistics

For all statistical analyses, confirm that the following items are present in the figure legend, table legend, main text, or Methods section.

n/a	Confirmed	
☐	☒	The exact sample size (*n*) for each experimental group/condition, given as a discrete number and unit of measurement
☐	☒	A statement on whether measurements were taken from distinct samples or whether the same sample was measured repeatedly
☐	☒	The statistical test(s) used AND whether they are one- or two-sided<br>*Only common tests should be described solely by name; describe more complex techniques in the Methods section.*
☒	☐	A description of all covariates tested
☐	☒	A description of any assumptions or corrections, such as tests of normality and adjustment for multiple comparisons
☐	☒	A full description of the statistical parameters including central tendency (e.g. means) or other basic estimates (e.g. regression coefficient) AND variation (e.g. standard deviation) or associated estimates of uncertainty (e.g. confidence intervals)
☐	☒	For null hypothesis testing, the test statistic (e.g. *F*, *t*, *r*) with confidence intervals, effect sizes, degrees of freedom and *P* value noted<br>*Give P values as exact values whenever suitable.*
☒	☐	For Bayesian analysis, information on the choice of priors and Markov chain Monte Carlo settings
☐	☒	For hierarchical and complex designs, identification of the appropriate level for tests and full reporting of outcomes
☐	☒	Estimates of effect sizes (e.g. Cohen's *d*, Pearson's *r*), indicating how they were calculated

Our web collection on statistics for biologists contains articles on many of the points above.

Software and code

Policy information about availability of computer code

Data collection	The proteomic raw data were performed by Q Exactive HF-X Mass Spectrometer, Thermo Fisher Scientific. The phosphoproteomic raw data were performed by Q Exactive HF-X Mass Spectrometer, Thermo Fisher Scientific. The whole exon sequencing data were obtained through, Illumina Novaseq 6000. Data of cell apoptosis and cell proliferation were collected on a Beckman Coulter flow cytometer (Beckman Coulter, Brea, CA, USA).
Data analysis	The data analysis was performed by programming language R (version 4.0.2) and GraphPad Prism (version 7.0). Most of them used broadly applied R packages and others used self-made R scripts according published papers: ConsensusClusterPlus (v1.50.0), pheatmap (v1.0.12) for supervised hierarchical clustering, Hmisc (v4.5-0) for spearman's correlation calculating, ggplot2 (v3.3.5) for scatter plot. For WES, BWA (v0.7.12, Li H et al.), SAMtools (v1.9, Li H et al.) and Picard (http://broadinstitute.github.io/picard/) were used to genome alignment, and muTect Software (Cibulskis K et al. 2013) was used for targeting Somatic SNV sites, and Strelka was used to test Somatic INDEL information. For RNA-Seq, raw data quality was assessed with the FastQC (v0.11.9) and the adaptor was trimmed with Trim_Galore (version 0.6.6). Reads were mapped by using STAR software (v2.7.7a). The mapped reads were assembled into transcripts or genes by using StringTie software (v2.1.4). Valid sequencing data was mapped to the reference human genome (UCSC hg19) by Burrows-Wheeler Aligner (BWA, v0.7.12) software to get the original mapping results stored in BAM format. SAMtools (v1.9) and Picard (http://broadinstitute.github.io/picard/) were used to sort BAM files and do duplicate marking, local realignment, and base quality recalibration to generate final BAM file for computation of the sequence coverage and depth. Somatic variants were then called, utilizing VarScan v2.3.8, MuTect v1.1.7) and InVEX (http://www.broadinstitute.org/software/invex/). SCNA analysis was performed by following somatic copy-number variation (CNV) calling pipeline in GATK's (GATK v 4.1.2.0) Best Practice. The results of this pipeline, segment files of every 1,000, were put in GISTIC2 (v2.0). Reads were mapped onto the human reference genome (GRCh38.p13 assembly) by using STAR software (v2.7.7a). The mapped reads were assembled into transcripts or genes by using StringTie software (v2.1.4) and the genome annotation file (hg38_ucsc.annotated.gtf). SCNAs affecting protein and phosphoprotein abundance in either "cis" (within the same aberrant locus) or "trans" (remote locus) mode were visualized using

"multiOmicsViz" (v1.18.0) R package. Kinase activity scores were inferred from phosphorylation sites by employing PTM signature enrichment analysis (PTM-SEA) using the PTM signatures database (PTMsigDB) v1.9.0 (https://github.com/broadinstitute/ssGSEA2.0). To investigate the impact of different biological processes pathway enrichment on immune clusters, the "GSVA" R package (v1.42.0) was used to conduct GSVA enrichment analysis. Standard statistical tests in this study were used to analyze the clinical data, including but not limited to Wilcoxon signed-rank test, Fisher's exact test, Kruskal-Wallis test. Flowjo version 10.7.1 (Becton Dickinson Life Science) was applied to analyze the cell apoptosis and cell proliferation through a Beckman Coulter flow cytometer (Beckman Coulter, Brea, CA, USA). The statistical significance of differences between two groups was calculated with the Wilcoxon rank-sum test; for more than two group comparisons, Kruskal–Wallis test was used. Fisher's exact test was used for categorical variables and Wilcoxon rank-sum test was used for continuous variables, when testing association of different groups with clinical variables. As for the correlation analysis between two proteins/phosphoproteins, Pearson's correlation (two-sided) of correlation coefficients were used. All statistical tests were two-sided except special explanation. To account for multiple-testing, the p values were adjusted using the Benjamini-Hochberg FDR correction. Data in the boxplot were presented median (central line), upper and lower quartiles (box limits), 1.5× interquartile range (whiskers). Statistical significance was considered when p-value < 0.05. For validation experiments, each was repeated at least three times independently, representative photos were shown.

For manuscripts utilizing custom algorithms or software that are central to the research but not yet described in published literature, software must be made available to editors and reviewers. We strongly encourage code deposition in a community repository (e.g. GitHub). See the Nature Portfolio guidelines for submitting code & software for further information.

Data

Policy information about availability of data

All manuscripts must include a data availability statement. This statement should provide the following information, where applicable:

- Accession codes, unique identifiers, or web links for publicly available datasets
- A description of any restrictions on data availability
- For clinical datasets or third party data, please ensure that the statement adheres to our policy

The proteome and phosphoproteome raw datasets and processed results files generalized in this study have been deposited to the ProteomeXchange Consortium (dataset identifier: PXD038867) via the iProX partner repository (https://www.iprox.cn/)126 under Project ID IPX0002184000. The VCF files of the WES data files were deposited to the European Genome-Phenome Archive (EGA) associated with the study EGAS00001006357 under project ID EGAD00001008987. The raw WES data are available in the Genome Sequence Archive127 (GSA) under restricted access HRA004048. The user can register and login to the GSA database website (https://ngdc.cncb.ac.cn/gsa-human/) and follow the guidance of "Request Data" to request the data step by step (https://ngdc.cncb.ac.cn/gsa-human/document/GSA-Human_Request_Guide_for_Users_us.pdf). The approximate response time for accession requests is about 2 weeks. The access authority can be obtained for Research Use Only. The user can also contact the corresponding author directly. Once access has been granted, the data will be available to download for 3 months. The gene expression profiles of DC cell lines in public dataset Expression 21Q2 in this study are available in the Depmap database (https://depmap.org/portal/download/?releasename=DepMap+Public+21Q2&filename=CCLE_expression.csv). The remaining data are available within the Article, Supplementary Information, or Source Data file. Source data are provided with this paper.

Human research participants

Policy information about studies involving human research participants and Sex and Gender in Research.

Reporting on sex and gender	A total of 156 duodenal cancer patients were enrolled in this study covering 438 tissue samples, in which 55 male and 101 female DC patients were included.
Population characteristics	A total of 438 samples were obtained from 156 duodenal cancer (DC) patients (gender: 55 males and 101 females, age range: 19–83 years, habit: 30 had drinking/smoking habit and 93 had no drinking/smoking habit (33 patients had no information of habits)), which were from 2012 to 2019 at Zhongshan hospital, Fudan University, and received no prior anti-cancer treatments.
Recruitment	A total of 156 duodenal cancer (DC) patients (gender: 55 males and 101 females, age range: 19–83 years, habit: 30 had drinking/smoking habit and 93 had no drinking/smoking habit (33 patients had no information of habits)) were randomly recruited from patients from 2012 to 2019 at Zhongshan hospital, Fudan University, and received no prior anti-cancer treatments.<br>There was no selection bias.
Ethics oversight	The present study was carried out in compliance with the ethical standards of Helsinki Declaration II and approved by the Institution Review Board of Fudan University Zhongshan Hospital (B2019-200R).

Note that full information on the approval of the study protocol must also be provided in the manuscript.

Field-specific reporting

Please select the one below that is the best fit for your research. If you are not sure, read the appropriate sections before making your selection.

☒ Life sciences ☐ Behavioural & social sciences ☐ Ecological, evolutionary & environmental sciences

For a reference copy of the document with all sections, see nature.com/documents/nr-reporting-summary-flat.pdf

Life sciences study design

All studies must disclose on these points even when the disclosure is negative.

Sample size	The proteomic profiling was performed on the 438 tissues samples from 156 duodenal cancer (DC) cases.<br>The phosphoproteomic analysis was performed on the 111 tissues samples from 49 DC cases.<br>The WES analysis was conducted on the 120 tissues samples from 47 DC cases.<br>No statistical method was used to predetermine sample size.<br>The functional and biological experiments were performed with at least three biological replicates to allow statistical significance testing through two-sided student's t-test.
Data exclusions	Two hundred early-stage DC (EDC) patients underwent ESD therapy from 2012 to 2019 at Zhongshan hospital, Fudan University, and received no prior anti-cancer treatments. Among of the EDC patients, 23 were precluded duo to the unavailability of their normal tissue samples, and 41 patients failed to pass the pathological quality check. Thus, 136 EDC patients were eligible for the establishment of the intended study cohort. In addition, 20 advanced-stage DC patients, without prior anticancer treatments, were randomly enrolled for the first visit from 2012 to 2019 at Zhongshan hospital, Fudan University. Therefore, a total of 156 DC patients were selected to construct DC cohort in this study.
Replication	All experiments were reliably reproduced and indicated in figure legends. The replicated analysis of 293T cell lysates were used for the quality control of the mass spectrometer.
Randomization	For multi-omic analysis, samples of DC patients were randomly divided into groups to avoid bias for protein/phosphoprotein quantification.
Blinding	The investigators who measured protein/phosphoprotein expression, WES data were blinded to patient information. The investigators who performed IHC were blinded to clinical information of duodenal cancer (DC) patients. For consensus clustering analyses, the investigators were blinded to group allocation during data collection.

Reporting for specific materials, systems and methods

We require information from authors about some types of materials, experimental systems and methods used in many studies. Here, indicate whether each material, system or method listed is relevant to your study. If you are not sure if a list item applies to your research, read the appropriate section before selecting a response.

Materials & experimental systems

n/a	Involved in the study
☐	☒ Antibodies
☐	☒ Eukaryotic cell lines
☒	☐ Palaeontology and archaeology
☐	☒ Animals and other organisms
☒	☐ Clinical data
☒	☐ Dual use research of concern

Methods

n/a	Involved in the study
☒	☐ ChIP-seq
☐	☒ Flow cytometry
☒	☐ MRI-based neuroimaging

Antibodies

Antibodies used	Anti-AARS1 (dilution 1:100, Proteintech, catalog No:17394-1-AP),<br>Anti-Poly (ADP-Ribose) polymer (dilution 1:100, Abcam, catalog No:14459),<br>Anti-Histone H2A.X (dilution 1:100, Cell Signaling Technology, catalog No: 7631),<br>Anti-Phospho-Histone H2A.X(Ser139) (dilution 1:200, Cell Signaling Technology, catalog No: 9718),<br>Anti-SARS1 (dilution 1:500, Abclonal, catalog No: A13350),<br>Anti--TARS1 (dilution 1:500, Abclonal, catalog No: A6993),<br>Anti-Flag (dilution 1:5000, Proteintech, catalog No: 20543-1-AP),<br>Anti-HA (dilution 1:5000, Proteintech, catalog No: 51064-2-AP),<br>Anti-PARP1 (dilution 1:500, Proteintech, catalog No: 13371-1-AP),<br>Anti-TARS1 (dilution 1:500, Proteintech, catalog No: 14773-1-AP),<br>Anti-GARS1 (dilution 1:500, Proteintech, catalog No: 15831-1-AP),<br>Anti-Actin (dilution 1:1000, Proteintech, catalog No: 66009-1-Ig)<br>Anti-GAPDH (dilution 1:5000, Proteintech, catalog No: 60004-1-Ig).
Validation	All antibodies used in this manuscript were obtained from the indicated commercial vendors and have been validated by the respective manufacturer, as described in their website.<br>Anti-AARS1 (dilution 1:100, Proteintech, catalog No:17394-1-AP) validated for immunohistochemistry (IHC) and western blotting by manufacturer [https://www.ptgcn.com/products/AARS-Antibody-17394-1-AP.htm],<br>Anti-poly(ADP-ribose) polymer (dilution 1:100, Abcam, catalog No:14459) validated for western blotting by manufacturer [https://www.abcam.cn/poly-adp-ribose-polymer-antibody-10h-ab14459.html],<br>Anti-Histone H2A.X (dilution 1:100, Cell Signaling Technology, catalog No: 7631) validated for western blotting by manufacturer [https://www.cellsignal.cn/products/primary-antibodies/histone-h2a-x-d17a3-xp-rabbit-mab/7631?site-search-

type=Products&N=4294956287&Ntt=h2ax&fromPage=plp],
Anti-Phospho-Histone H2A.X(Ser139) (dilution 1:200, Cell Signaling Technology, catalog No: 9718) validated for western blotting by manufacturer [https://www.cellsignal.cn/products/primary-antibodies/phospho-histone-h2a-x-ser139-20e3-rabbit-mab/9718?site-search-type=Products&N=4294956287&Ntt=h2ax&fromPage=plp],
Anti-SARS1 (dilution 1:500, Abclonal, catalog No: A13350) validated for western blotting by manufacturer [https://abclonal.com.cn/catalog/A13350],
Anti--TARS1 (dilution 1:500, Abclonal, catalog No: A6993) validated for western blotting by manufacturer [https://abclonal.com.cn/catalog/A6993],
Anti-Flag (dilution 1:5000, Proteintech, catalog No: 20543-1-AP) validated for western blotting by manufacturer [https://www.ptgcn.com/products/Flag-Tag-Antibody-20543-1-AP.htm],
Anti-HA (dilution 1:5000, Proteintech, catalog No: 51064-2-AP) validated for western blotting by manufacturer [http://www.ptgcn.com/products/HA-tag-Antibody-51064-2-AP.htm],
Anti-PARP1 (dilution 1:500, Proteintech, catalog No: 13371-1-AP) validated for western blotting by manufacturer [https://www.ptgcn.com/products/PARP1-Antibody-13371-1-AP.htm]
Anti-TARS1 (dilution 1:500, Proteintech, catalog No: 14773-1-AP) validated for western blotting by manufacturer [https://www.ptgcn.com/products/TARS-Antibody-14773-1-AP.htm],
Anti-GARS1 (dilution 1:500, Proteintech, catalog No: 15831-1-AP) validated for western blotting by manufacturer [https://www.ptgcn.com/products/GARS-Antibody-15831-1-AP.htm],
Anti-Actin (dilution 1:1000, Proteintech, catalog No: 66009-1-Ig) validated for western blotting by manufacturer [https://www.ptgcn.com/products/Pan-Actin-Antibody-66009-1-Ig.htm],
Anti-GAPDH (dilution 1:5000, Proteintech, catalog No: 60004-1-Ig) validated for western blotting by manufacturer [https://www.ptgcn.com/products/GAPDH-Antibody-60004-1-Ig.htm].

Eukaryotic cell lines

Policy information about cell lines and Sex and Gender in Research

Cell line source(s)	Human HEK293T (ATCC, CRL-11268; RRID: CVCL_QW54) cell; Human Hutu80 (ATCC, CRL-7928; RRID: CVCL_1301); WDC-1 (Cobioer, CBP61181; RRID: CVCL_R803)
Authentication	All the cell lines were authenticated with short tandem repeat (STR) profiling method.
Mycoplasma contamination	All cell lines were tested negative for mycoplasma contamination.
Commonly misidentified lines (See ICLAC register)	No commonly misidentified cell lines were used in the study.

Animals and other research organisms

Policy information about studies involving animals; ARRIVE guidelines recommended for reporting animal research, and Sex and Gender in Research

Laboratory animals	Four- to six-week-old male BALB/c nude mice were obtained from Shanghai SLAC Laboratory Animal Co., Ltd. (Shanghai, China) for in vivo xenografts (n = 6 mice per group). Mice were housed in polycarbonate cages, and provided free access to food and water with a 12-h light:dark cycle.
Wild animals	No wild animals were involved.
Reporting on sex	A total of 36 BALB/c nude mice (male) were obtained in this study.
Field-collected samples	No field-collected samples were involved.
Ethics oversight	All experimental procedures involving animals were approved by the Fudan University Institutional Animal Care and Use Committee and were conducted in accord with the National Institutes of Health Guidelines for the Care and Use of Laboratory Animals. The maximal tumor burden permitted by the committee is 2000 mm3.

Note that full information on the approval of the study protocol must also be provided in the manuscript.

Flow Cytometry

Plots

Confirm that:

☒ The axis labels state the marker and fluorochrome used (e.g. CD4-FITC).

☒ The axis scales are clearly visible. Include numbers along axes only for bottom left plot of group (a 'group' is an analysis of identical markers).

☒ All plots are contour plots with outliers or pseudocolor plots.

☒ A numerical value for number of cells or percentage (with statistics) is provided.

Methodology

Sample preparation	Cell lines were routinely cultured prior to death induction. Samples were filtered prior to staining and kept on ice during staining. A Beckman Coulter flow cytometer (Beckman Coulter, Brea, CA, USA) was used to detect apoptotic cells. An Annexin V-FITC Apoptosis Detection Kit (BD Biosciences) was used to detect apoptotic cells according to manufacturers' instruction.
Instrument	Data were collected on a Beckman Coulter flow cytometer (Beckman Coulter, Brea, CA, USA).
Software	Flowjo version 10.7.1 (Becton Dickinson Life Science)
Cell population abundance	Purity of isolated samples was obtained by antibody stain and FACS. Sample purity was greater than 95%.
Gating strategy	Apoptosis of cells were gated using Annexin V and PI following doublet exclusion using FSCHxW and SSC-HxW.

☒ Tick this box to confirm that a figure exemplifying the gating strategy is provided in the Supplementary Information.
